# Cooperation with Teachers as a Mediator of the Relationship between Family Conflict and Children’s Psychological Difficulties

**DOI:** 10.3390/ijerph192013151

**Published:** 2022-10-13

**Authors:** Marcella Caputi, Barbara Forresi, Ludovica Giani, Simona Scaini

**Affiliations:** 1 Department of Life Sciences, University of Trieste, Via E. Weiss 21, 34128 Trieste, Italy; 2 Child and Youth Lab, Sigmund Freud University of Milan, Via Ripa di Porta Ticinese 77, 20143 Milan, Italy

**Keywords:** conflict, parent–child relationship, child behavior, mediation, cooperation, school, teachers, internalizing symptoms, externalizing symptoms, psychopathology

## Abstract

Parent–child conflict can have a series of negative consequences concerning youth emotional and behavioral development. The link between family conflict and children’s externalizing symptoms is well established, whereas the association with internalizing symptoms is less explored. Within the school context, children are engaged in other meaningful relationships (i.e., with teachers and peers) which contribute to their growth. This cross-sectional study aimed at understanding whether a cooperative behavior with the teachers is able to mediate the association between parent–child conflict and children’s psychopathological symptoms. We recruited 319 (150 boys) school-aged children (M = 11.3 years; SD = 1.8 years) and their parents and teachers. Children self-reported on their internalizing symptoms, whereas parents completed a questionnaire concerning their relationship with the child, and teachers rated children’s behavior and internalizing/externalizing symptoms. Analyses conducted through Hayes’ PROCESS tool showed that cooperation with the teacher partially mediated the association between parent–child conflict and child-reported depressive symptoms. Notably, cooperative behavior fully mediated the link between parent–child conflict and children’s internalizing and externalizing symptoms reported by teachers. Difficulties exhibited at school partly derive from a conflictual home environment. Our findings showed that such problems can be reduced thanks to a cooperative relationship with the teacher.

## 1. Introduction

The parent–child relationship is the first and most important bond that individuals develop and is likely to greatly impact youth socioemotional development [1,2,3,4,5]. The quality of the parent–child relationship is also significantly associated with child internalizing/externalizing difficulties [6,7,8]. Therefore, investigating the quality of such a relationship appears to be crucial to understand subsequent interpersonal relationships and behaviors manifested in different social contexts [9,10]. While a warm and supportive bond between a parent and his/her child favors a healthy socioemotional development [11,12], a conflictual relationship facilitates dysfunctionality in further interactions [13]. Parent–child relationships can be considered conflictual when both members of the dyad display negative affect and behaviors, which make the interaction difficult. It follows that when exchanges in the family are characterized by elevated rates of conflict, children and adolescents are more at risk of developing mental health issues both in the short- and long-term [14,15,16,17,18]. This is particularly relevant during stressful times, such as the COVID-19 period [19,20,21]. According to a recent study [22], parent–child conflict was highly frequent in families (77.9%) during COVID-19 pandemic, with 31.4% of families reporting an increase in conflict, compared with the previous year. These conflicts were mostly due to school difficulties, daily arrangements, and the use of digital technologies.

### 1.1. Parent–Child Conflict and Externalizing Problems

Gorman-Smith and colleagues [23] highlighted that antisocial behaviors can be both maintained and reinforced by parent–child conflict. In particular, mother- and child-reported conflict appeared to account for the development of ADHD, conduct disorder, and oppositional defiant disorder (in comorbidity) among Caucasian children aged 11 years old [14]. In their cross-sectional study, El-Sheikh and Elmore-Staton [24] found that mother–child conflict partially mediated the association between interparental conflict and internalizing difficulties in school-age children from Caucasian middle-class families. Moreover, high rates of conflictual parent–child relationships predicted several behavioral difficulties, acting as a model for aggressive and delinquent behaviors for youth coming from different socioeconomic environments [25,26]. More specifically, in a sample of low-income families, higher conflict between mother and son aged 5–6 predicted moderately increasing antisocial behavior in one group and high but decreasing antisociality in another group from ages 5 to 11 [25]. These findings consistently show a predictive role played by parent–child conflict on children’s behavioral problems, particularly disruptive behavior.

### 1.2. Parent–Child Conflict and Internalizing Problems

As most research has targeted externalizing difficulties, there is a high need for studies addressing the link between parent–child conflict in early stages of development and children’s internalizing difficulties, e.g., [14,26], which is less clear.

In a study by Branje et al. [27], higher levels of depressive symptoms among adolescents were associated with lower mother–child relationship quality in the study, while lower father–child relationship quality predicted depressive symptoms for boys only.

Sentse and Laird [28] longitudinally investigated the impact of parent–child conflict, finding that it was related to both antisocial and depressive symptoms one year later. In a sample of white adoptive families, Klahr et al. [17] found that parent–child conflict predicted the development of conduct problems four years later, but conduct problems did not predict increased parent–child conflict, suggesting that the parent–child relationship is the driving force in the emergence of conduct problems, and not the opposite. Furthermore, according to Trentacosta et al. [29] four distinct trajectories of mother–son conflict from middle childhood to adolescence could be identified in a sample of at risk, ethnically diverse families: high-stable, high-decreasing, moderate, and low conflict. Compared to the moderate and low-conflict groups, the chronically high and high-decreasing showed higher rates of antisocial behaviors at age 15, suggesting that changes over time in conflict differentially impact adolescent problem behavior. Taken together, the works of Burt et al. [14], Klahr et al. [17], and Trentacosta et al. [29] suggest that the presence of high levels of parent–child conflict during the school-age period and early adolescence is related to later child behavior problems.

### 1.3. Conceptual Framework

Children who face prolonged parent–child conflict are more likely to develop either internalizing or externalizing symptoms [30], whereas the engagement in supportive and cooperative relationships may act as a protective factor against the risk of impairments in the child’s socioemotional development [11,12].

According to the differential susceptibility model, some individuals are disproportionately vulnerable to both positive and negative developmental experiences and environmental adversities [31,32]. Therefore, many of those whom the diathesis-stress framework considered genetically more prone to be adversely affected by negative life experiences may also be likely to benefit from supportive and enriching ones. The level of vantage sensitivity depends on the presence of promoting factors, such as high-quality child care, prosocial behavior, supportive relational networks, sensitive parenting, and good academic achievement [33]. Based on these theoretical premises, being aware of factors that might decrease the effects of potential triggers for psychopathology is crucial for enhancing child adjustment chances.

### 1.4. The Present Study

While several mediating factors in the relationship between parent–child conflict and children’s internalizing and externalizing difficulties have been explored [24,34,35], only a few studies explored the role of the relationship with school teachers. Nonetheless, teachers and peers represent important dimensions for children’s social–emotional adjustment [36]: thanks to everyday interaction, they may help them build trusted and healthy relationships in the school environment, understanding their emotions and behaviors in classroom. Teachers can also model how to deal with conflicts in a constructive way and help develop social competences such as cooperation. Not surprisingly, Acar and colleagues [37] showed that children have lower behavioral regulation skills when they experience a combination of low child–parent closeness and high child–teacher conflict. Another study showed that parent–child conflict was a risk factor for aggression in female adolescents, while teacher support was a protective factor [38].

Within this research study, we investigated parent–child conflict at home and children’s behavior at school, gathering information from parents and teachers. The exploration of the relational pattern among these constructs can shed light on how the crucial bonds with parents and teachers contribute to shaping children’s functioning in middle childhood. Specifically, we hypothesize a positive link between parent–child conflict and children’s internalizing/externalizing symptoms manifested at school. We also predict that this link is mediated by the child’s ability and willingness to cooperate with the teacher.

## 2. Materials and Methods

### 2.1. Participants

A sample of 368 children was recruited from four elementary schools and four middle schools randomly selected in one large and two medium-sized cities of Northern Italy. Following a complete description of the research, we obtained school principals’ and teachers’ approval for our study. Our target were children attending the third and the fifth year of primary school and the second year of middle school. Parents of all children attending these classes were invited to participate in the research. Parents of 326 children accepted to sign the informed consent and children gave oral assent. Nonetheless, seven children were excluded due to insufficient data available. Therefore, the final sample was composed of 319 children (150 boys; 47%) attending the third (*n* = 84) and the fifth (*n* = 70) year of primary school and the second (*n* = 165) year of middle school (M = 11 years and 3 months; SD = 1 year and 8 months). No child was affected by neurological/psychiatric disorders.

### 2.2. Procedure

Children completed a self-report questionnaire on depressive symptoms at school during a collective testing session. Two teachers in every classroom jointly completed, for each student, two questionnaires on children’s social skills and behavior, whereas parents received an envelope containing an informed consent form and one questionnaire on the relationship with their child to be filled out at home and brought back to the school.

### 2.3. Ethical Statement

Ethics approval was not mandatory for this research. Nonetheless, the project was run in accordance with the ethical standards of the Declaration of Helsinki and informed consent was obtained from participants.

### 2.4. Measures

Children completed the Children’s Depression Inventory (CDI) [39], which is a 27-item self-report measure assessing children and adolescents’ depressive and dysthymic symptoms. For every item, children were asked to choose the statement—out of three reflecting gradual symptoms of severity—that best described their mood in the past two weeks (score range = 0–54). Internal consistency for this measure was good (Cronbach’s alpha = 0.84). The psychometric properties of the Italian version of the CDI have been validated in previous research [40].

Teachers evaluated children’s social skills using the teacher form of the Social Skills Rating System (SSRS) [41], which consists of 30 items, each rated on a 3-point Likert scale ranging from 0 (never) to 2 (often). Three types of behavior were considered: Assertion (e.g., initiates conversations, makes friends easily), Cooperation (e.g., “Finishes class assignments on time”), and Self-control (e.g., “Controls temper in conflicts with peers”). Each subscale was composed of 10 items (score range for each subscale: 0–20). Internal consistency of the three subscales was 0.86, 0.92, and 0.91, respectively.

Moreover, teachers evaluated children’s behavior through the Strengths and Difficulties Questionnaire (SDQ) [42], which is a 25-item questionnaire assessing Emotional Symptoms, Peer Problems, Conduct Problems, Hyperactivity/Inattention, and Prosocial Behavior (5 items for each subscale). For each item (e.g., “Rather solitary, tends to play alone”), responses are given on a Likert scale ranging from 0 (not true) to 2 (certainly true). Following the scoring procedure, we summed Emotional Symptoms and Peer Problems scores to obtain an Internalizing Problems score (range: 0–20), and summed Conduct Problems and Hyperactivity/Inattention scores to obtain an Externalizing Problems score (range: 0–20). For our sample, the Cronbach’s alphas for Internalizing and Externalizing Problems subscales were 0.80 and 0.85, respectively. The psychometric properties of the Italian version of the SDQ for teachers have been validated in previous research [43].

Parents completed the Child–Parent Relationship Scale-Short Form (CPRS-SF) [44]. This is a 15-item questionnaire on a 5-point Likert scale, ranging from 1 (Definitely does not apply) to 5 (Definitely applies). Items are grouped into two dimensions: Closeness and Conflict. The Closeness subscale (7 items) evaluates positive aspects of the relationship (score range: 7–35); the Conflict subscale (8 items) evaluates the degree of conflicts and disagreements (score range: 8–40). Internal reliability of the subscales in the present study was good (Cronbach’s alpha = 0.80 for Closeness and 0.83 for Conflict). The Italian version of the CPRS has been used in previous research in its long form, with similar psychometric indices [2].

### 2.5. Data Analysis Plan

We first present descriptive statistics to allow for comparisons with past and future studies using the same variables. We then present *t*-tests conducted to explore gender differences. Next, we present correlations among the study variables, which guided our core analyses. Finally, we present mediation analyses to describe specific relationships among parent–child conflict, student–teacher cooperation and children’s internalizing and externalizing symptoms. Specifically, in order to test the relationship among these variables, we assumed a simple mediation, described by Hayes [45] as model 4 of mediation. In this model, the independent variable X influences the dependent variable Y with an indirect effect via the mediator M. The indirect effect of X on Y mediated by M is calculated by the sum of the effect of X on M and the effect of M on Y, controlling for X. We tested three separate models. In all of them, Child–parent Conflict was the independent variable (X) and Cooperation with the teacher represented the mediator (M). The dependent variable (Y) was different in every model: in the first one, it was represented by children’s externalizing problems reported by teachers, in the second one it was represented by child-reported depressive symptoms, and in the third one it was represented by internalizing problems reported by teachers. In all models, we used the PROCESS macro model 4 [45] to calculate the mediation effect, with 5000 bootstrap estimates for the construction of 95% bias-corrected confidence intervals.

## 3. Results

### 3.1. Preliminary Analyses

Independent-samples *t*-tests showed significant gender differences only in children’s Externalizing Problems and Cooperation with the teacher (see Table 1).

As shown in Table 2, parent–child conflict was positively correlated with children’s’ internalizing and externalizing problems reported by teachers and also with children’s self-reported depressive symptoms. Moreover, parent–child conflict was negatively correlated with parent–child closeness and with cooperation with the teacher and self-control. On the other hand, parent–child closeness was positively correlated with cooperation and assertion, and negatively correlated with externalizing problems.

### 3.2. Main Analyses

**Figure 1:**
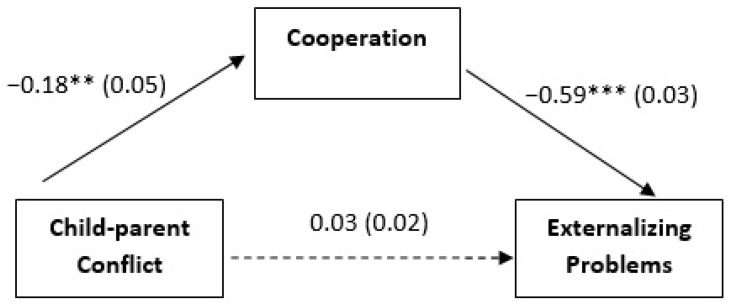
Cooperation as mediator of the association between Child–parent Conflict and Externalizing Problems. Unstandardized coefficients are reported with standard errors in parentheses. The model includes Gender as a covariate of both Cooperation and Externalizing Symptoms. Analyses were based on 5000 bootstrap samples with 95% bias-corrected confidence intervals; ** *p* < 0.01; *** *p* < 0.001.

**Figure 2:**
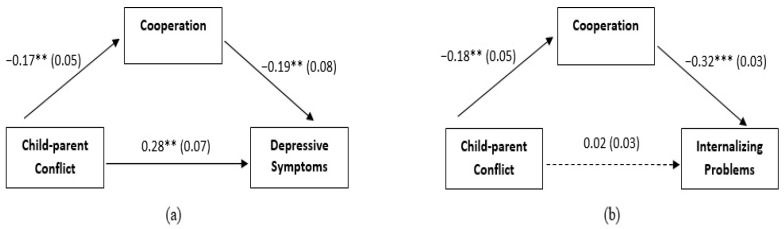
Cooperation as mediator of the association between Child–parent Conflict and Depressive Symptoms (**a**) and Internalizing Problems (**b**). Unstandardized coefficients are reported with standard errors in parentheses. Both models include Gender as a covariate of Cooperation. Analyses were based on 5000 bootstrap samples with 95% bias-corrected confidence intervals; ** *p* < 0.01; *** *p* < 0.001.

Following our data analysis plan, we tested three separate mediation models. In our first model (see Figure 1), Child–parent Conflict was the independent variable (X), Externalizing Problems were the dependent variable (Y), and Cooperation with the teacher represented the mediator (M). In our second model (see Figure 2a), Child–parent Conflict was the independent variable (X), Depressive Symptoms were the dependent variable (Y), and Cooperation with the teacher represented the mediator (M). In our third model (see Figure 2b), Child–parent Conflict was the independent variable (X), Internalizing Problems were the dependent variable (Y), and Cooperation with the teacher represented the mediator (M).

In the first model, the total effect was significant (effect = 0.1384, SE = 0.0371, 95% CI [0.0653, 0.2115]), direct effect of Child–parent Conflict on Cooperation with the teacher (effect = −0.1792, SE = 0.0476, 95% CI [−0.2728, −0.0856]), and Cooperation with the teacher on Externalizing Problems (effect = −0.5882, SE = 0.0292, 95% CI [−0.6456, −0.5308]) were significant. Moreover, gender was included in the model as a covariate based on *t*-tests: it had a significant effect on Cooperation = 2.4127, SE = 0.5079, 95% CI [1.4134, 3.4120] but not on Externalizing Problems = −0.5146, SE = 0.2706, 95% CI [−1.0470, 0.0178]. Nonetheless, the direct effect of Child–parent Conflict on Externalizing Problems became non-significant after controlling for Cooperation (effect = 0.0329, SE = 0.0250, 95% CI [−0.0163, 0.0822]). The indirect effect of Child–parent Conflict on Externalizing Problems through Cooperation was significant (effect = 0.1054, SE = 0.0293, 95% CI [0.0497, 0.1653]).

However, in the second model, all the paths were significant (total effect of Child–parent Conflict on Depressive Symptoms = 0.3094, SE = 0.0672, 95% CI [0.1771, 0.4417], effect of Child–parent Conflict on Cooperation with the teacher = −0.1705, SE = 0.0490, 95% CI [−0.2669, −0.0742], effect of Cooperation with the teacher on Depressive Symptoms = −0.1863, SE = 0.0764, 95% CI [−0.3366, −0.0360]. Moreover, gender was included in the model as a covariate based on *t*-tests: it had a significant effect on Cooperation = 2.4222, SE = 0.5237, 95% CI [1.3916, 3.4259]. Finally, the effect of Child–parent Conflict on Depressive Symptoms was reduced but still significant (direct effect = 0.2782, SE = 0.0679, 95% CI [0.1445, 0.4118]) after controlling for Cooperation with the teacher (indirect effect = 0.0318, SE = 0.0146, 95% CI [0.0089, 0.0685]). In other words, Cooperation with the teacher partially mediated the relation between Child–parent Conflict and Depressive Symptoms. In the third model, the total effect of Child–parent Conflict on Internalizing Problems (effect = 0.0818, SE = 0.0331, 95% CI [.0168, 0.1469]), the direct effect of Child–parent Conflict on Cooperation with the teacher (effect = −0.1792, SE = 0.0476, 95% CI [−0.2728, −0.0856]), and of Cooperation with the teacher on Internalizing Problems (effect = −0.3231, SE = 0.0334, 95% CI [−0.3889, −0.2574]) were significant. Moreover, gender was included in the model as a covariate based on *t*-tests: it had a significant effect on Cooperation = 2.4127, SE = 0.5079, 95% CI [1.4134, 3.4120]. Nonetheless, the direct effect of Child–parent Conflict on Internalizing Problems became non-significant after controlling for Cooperation (effect = 0.0247, SE = 0.0296, 95% CI [−0.0336, 0.0830]), whereas the indirect effect of Child–parent Conflict on Internalizing Problems through Cooperation was significant (effect = 0.0579, SE = 0.0170, 95% CI [0.0265, 0.0934]). In other words, high conflict perceived by parents in their relationship with the child reduces the ability of the child to cooperate with adults in the school setting that, in turn, increases the likelihood of the child to develop and exhibit Internalizing Problems.

## 4. Discussion

The primary aim of the current study was to investigate the relationship between parent–child conflict and the child’s behavior at school and the mediational role of the child’s ability and willingness to cooperate with the teacher.

Several studies in the literature indicated the beneficial effects of a positive parent–child relationship and academic performance, social, and emotional adjustment at school [1,2,5,33,46]. Only a few previous studies explored the role of the relationship with school teachers, e.g., [37,47].

In the first part of the present study, we analyzed the role of cooperation with the teacher in mediating the relation between child–parent conflict on depressive symptoms referred by the child. Our data seem to indicate that mediation is partial: conflict with parents is linked to depressive symptoms independently from cooperation with the teacher. However, the indirect effect of the conflict on depressive symptoms (which passes through the cooperation) is also significant, meaning that special attention should be paid to this aspect. In fact, a lack of cooperation with the teacher, which might be related to the conflict with the adult/parental figures, could exacerbate internalizing symptoms. At the same time, since the indirect effect is significant, cooperation with the teacher could reduce depressive symptoms arising from a conflict in the family.

When we repeated the model looking at externalizing problems as an outcome, we found a complete mediation effect of cooperation, meaning that in itself a parent–child conflict is not tightly related to externalizing behavior at school: the link between conflict and externalizing symptomatology becomes significant when the child does not cooperate with the teacher. The data seems to show that cooperation with the teacher is crucial, because—if missing—it can amplify the damaging consequences of a conflictual domestic relationship. These results confirm that there is an association between parent–child conflict and social competence [30,48] and that a family conflict might be particularly dangerous for children when they are not provided with the right environment to learn cooperation skills with adults. The lack of these skills is immediately evident in the school context and can be related to unhealthy competitive behaviors, opposition, and conflicts. The scenario in which a child lives in a conflictual environment both at home (with parents) and at school (with teachers) is clearly the worst from a developmental point of view, given the presence of dysfunctional relationships with caregivers and meaningful adults with an educational role in a delicate phase of development and exchange with adults (middle childhood). The quality of their first relationships with parents and teachers help them to adjust successfully to society. The role of conflict with parents and teachers in the acquisition of lower behavioral regulation skills in children is therefore confirmed [37].

It is also very interesting that results showed significant gender differences only in children’s Externalizing Problems and Cooperation with the teacher. This finding is coherent with the literature, which reports a heightened prevalence rate of externalizing symptoms, particularly hyperactivity, inattention, and aggression, among boys from the general population [49,50,51]. Girls are theorized to be comparably free of externalizing problems during early-to-middle childhood due to biological, cognitive, and social factors [52]. Likewise, higher levels of cooperative behaviors are usually observed in girls [53]. Nevertheless, the levels of cooperation in boys and girls have been shown to vary significantly according to the presence of observers [54]. Thus, the role of cooperation skills with adults are not to be underestimated and teachers should be provided with specific instruments to train children’s cooperation. In addition, teachers should keep in mind that each student might show different levels of cooperation and that the level of cooperation may vary during the school year. Specific school programs might help children to learn cooperative skills that can be used both at school and outside the school context.

Correlations also showed that children having a close relationship with the parents are judged by the teachers as assertive and cooperative. However, the strongest (negative) association was between conflict and cooperation. In conclusion, children who present both externalizing and internalizing symptomatology at school should be monitored, because both these problems could arise from a family conflict and could be reduced by the modeling and protective role of a good relationship with the teacher.

Our findings confirm the importance of healthy relationships between children and meaningful adults, both parents and teachers, in order for them to develop socially competent skills (including cooperation, assertiveness and self-regulation) [37]. Relationships with adults are crucial for children’s socio-emotional development: not surprisingly, some authors [47] have recently highlighted the role of the co-caring relationship, and the need for a co-caring framework as a guide for promoting parent–teacher relationships.

Indeed, the present research highlights the need to invest more in the family–school relationship and has several implications for both clinical work with families of school-aged children and for training interventions with teachers and educators in the school context. School plays a crucial role in fostering mental health and resilience in childhood, and the quality of the student–teacher relationship is particularly important for children without supportive family environments.

In addition, in a preventive perspective, our data indicate the need of including the use of assertiveness by teachers, as this crucial skill fosters cooperation, acting as a further protective factor. On the other hand, schools could implement training for children aimed at increasing their soft skills in order to better manage relationships with peers and adults.

## 5. Conclusions

Although the current study has several strengths, its findings should be interpreted within the context of some limitations.

First of all, we considered overall parent–child conflict, without differentiating between mother–child and father–child relationships. Analyzing these differences could lead to alternative scenarios, depending on the parent involved in the conflict.

Secondly, the design was correlational. A longitudinal design would have allowed us to investigate the trajectories of the relationships over time. In addition, a sequential design could shed light on potential differences among distinct age groups, to understand whether conflict with parents is more deleterious in certain phases or whether the levels of cooperation with the teacher are particularly low in some school years. Exploring continuity in parent–child conflicts is extremely important, in order to better understand its impact on children’s development. Still very little is known about how parent–child conflict changes during children’s development, and how it impacts the development of emotional and behavioral problems. In their notable study, Driscoll and Pianta [44] observed that conflict was higher during preschool than in school age, suggesting a decline over time. Moreover, mothers reported higher levels of conflict than fathers [44]. Investigating the patterns of cooperation in the light of parent–child interactions would be enlightening to see if, and to what extent, cooperation with other meaningful adults is possible, even in the presence of highly conflictual relationships. A collaborative way of managing conflicts could indeed influence the associations among the variables of interest [55].

Thirdly, teachers answered questionnaires about students’ emotional/behavioral problems (SDQ) and social skills (SSRS). Although other measures were filled by children and parents, this could have promoted a sort of bias in children’s classification (it is likely, for example, that a child classified as “cooperant” or “self-controlled” is also described as better behaved).

Moreover, even if gender was included as a covariate in mediation models, we did not control for other demographic variables that could have affected the links among the variables. Further studies could indeed explore age differences or gather other information from families (such as socioeconomic status) or differentiate between school types (e.g., public vs. private).

Finally, future studies could also explore whether higher cooperative behaviors are perceived by the teacher when there is a good cooperation between parents and teachers. Notably, researchers interested in contingencies of effects might decide to perform moderation analyses instead of mediation analyses. Indeed, the present study explored the role of cooperation as a mechanism by which conflict acts on symptoms display. However, understanding *when* that effect exists or not, and in what magnitude, is also extremely important.

## Figures and Tables

**Table 1:** Descriptive statistics and *t*-tests for gender differences in the study variables.

Variable	M Mean (SD)	F Mean (SD)	t (df)	*p*	Cohen’s d
Depressive Symptoms	10.27 (6.533)	11.53 (6.282)	−1.714 (302)	0.088	0.017
Cooperation	13.47 (4.605)	15.90 (4.537)	−4.724 (315)	<0.001	0.053
Assertion	13.99 (2.783)	13.53 (2.817)	1.466 (310)	0.144	0.478
Self-control	13.39 (3.398)	13.56 (3.263)	−0.452 (310)	0.652	0.005
Internalizing Problems	3.18 (3.232)	3.05 (3.076)	0.377 (315)	0.707	0.004
Externalizing Problems	4.81 (4.028)	2.88 (3.105)	4.751 (276.97)	<0.001	0.054
Child–parent Closeness	26.973 (4.439)	27.458 (4.438)	−0.970 (314)	0.333	0.011
Child–parent Conflict	16.668 (5.175)	16.815 (5.471)	−0.245 (314)	0.807	0.003

**Table 2:** Correlations among the study variables.

	2	3	4	5	6	7	8
1. Depr. Symptoms	−0.183 **	−0.059	−0.098	0.122 *	0.223 ***	0.036	0.259 ***
2. Cooperation	-	0.416 ***	0.694 ***	−0.497 ***	−0.779 ***	0.164 **	−0.199 ***
3. Assertion		-	0.538 ***	−0.438 ***	−0.326 ***	0.156 **	−0.069
4. Self-control			-	−0.445 ***	−0.679 ***	0.099	−0.137 *
5. Int. Problems				-	0.409 ***	−0.047	0.139 *
6. Ext. Problems					-	−0.144 *	0.197 ***
7. C–p Closeness						-	−0.252 ***
8. C–p Conflict							-

Note. Significance levels * *p* ˂ 0.05, ** *p* ˂ 0.01, *** *p* ˂ 0.001; Depr. Symptoms = Depressive symptoms investigated with the Children’s Depression Inventory (CDI); Int. Problems = Internalizing Problems (subscale of the Strengths and Difficulties Questionnaire); Ext. Problems = Externalizing Problems (subscale of the Strengths and Difficulties Questionnaire); C–p Closeness = Child–parent Closeness (subscale of the Child–Parent Relationship Scale-Short Form); C–p Conflict = Child–parent Conflict (subscale of the Child–Parent Relationship Scale-Short Form).

## Data Availability

Data may be obtained from the first author upon reasonable request.
